# Assessing the Impact of Oral Isotretinoin on the Menstrual Cycle: A Prospective Study on Predictors of Menstrual Irregularities

**DOI:** 10.3390/medicina60050832

**Published:** 2024-05-19

**Authors:** Diala Alshiyab, Haitham Bassam Marie, Raghad Alrawashdeh, Nour Alrawashdeh, Yaman B. Ahmed, Ausama Atwan

**Affiliations:** 1 Department of Dermatology, King Abdullah University Hospital, Faculty of Medicine, Jordan University of Science and Technology, P.O. Box 3030, Irbid 22110, Jordan; haithambmarie@gmail.com; 2 Faculty of Medicine, Yarmouk University, Irbid 21163, Jordan; 2019999134@ses.yu.edu.jo; 3 Faculty of Medicine, Jordan University of Science and Technology, Irbid 22110, Jordan; nwalrawashdeh19@med.just.edu.jo (N.A.); ybahmed180@med.just.edu.jo (Y.B.A.); 4 Department of Dermatology, Cardiff University, Cardiff CF10 3XB, UK; abouatwanaa@cardiff.ac.uk

**Keywords:** isotretinoin, menstruation, amenorrhea, menstrual cycle, change in menses

## Abstract

*Background and Objectives*: This study aims to evaluate the association between the use of oral isotretinoin and menstrual irregularities in acne patients with previously regular menstrual cycles. *Materials and Methods*: A prospective observational study was conducted on 58,599 female patients aged 14 to 36 at King Abdullah University Hospital in Irbid, Jordan. The patients were followed for a period of 4.5 to 8 months during treatment and for 2 months post-treatment. Menstrual cycle changes were documented, and statistical analysis was performed to identify any significant associations. *Results*: A total of 111 (37.1%) patients, who were previously known to have regular menstrual cycles, complained of menstrual changes while using oral isotretinoin. Ninety-nine of those patients who complained of menstrual changes had their cycles back to normal post-treatment. There is a significant difference in the total accumulative dose between those with changes in menses and those without; *p*-value [0.008]. The most common change that occurred was amenorrhea (*p* < 0.001), followed by oligomenorrhea and menorrhagia (*p* < 0.001 and *p* = 0.050, respectively). The duration of treatment was a significant predictor of menstrual irregularities, with an odds ratio (OR) of 5.106 (95% CI: 1.371–19.020, *p* = 0.015), indicating a higher likelihood of menstrual changes with increased treatment duration. The total accumulative dose was also significantly associated with menstrual irregularities (OR = 0.964; 95% CI: 0.939–0.990; *p* = 0.006). Additionally, a family history of PCOS significantly increased the odds of menstrual irregularities (OR = 3.783; 95% CI: 1.314–10.892; *p* = 0.014). *Conclusions*: The study identified that 37.1% of the participants experienced changes in their menstrual cycles while undergoing isotretinoin therapy, with the vast majority (89.2%) returning to normal within two months post-treatment. Our logistic regression analysis pinpointed the duration of isotretinoin treatment, the total accumulative dose, and a family history of PCOS as significant predictors of menstrual irregularities.

## 1. Introduction

Oral isotretinoin, a synthetic vitamin A derivative, is a potent medication approved for the treatment of moderate acne resistant to conventional therapies or that relapses frequently after finishing oral antibiotics, as well as severe nodulocystic acne [1]. It works by inhibiting the growth of propionibacterium and lowering sebum production, thereby decreasing the formation of comedones by reducing hyperkeratinization through an unknown mechanism [2]. It is routinely prescribed for 4–6 months at a dosage of 0.5–2 mg/kg/day. Like other retinoids, isotretinoin is known for having multiple side effects that cannot be ignored, some of which are significantly tragic if they happen. These side effects include teratogenicity [3], myalgia, arthralgia, depression, hyperlipidemia, and mucocutaneous dryness. Menstrual irregularities in women of childbearing age have been documented in several studies [4,5,6], which mostly resolve once the medication is discontinued. Despite this, menstrual irregularities are not listed as a side effect in the patient introductory brochure for iPLEDGE, the risk management program mandated by the U.S. The underreporting of menstrual irregularities among oral isotretinoin users in Jordan presents a significant need for understanding the drug’s side effects, highlighting a crucial area for further research and clinical attention. In an attempt to fill this gap, we aimed to shed light on the relationship between isotretinoin and irregular menses to provide evidence-based guidelines for dermatologists prescribing isotretinoin and to identify potential strategies for managing this side effect.

## 2. Materials and Methods

This is a prospective single-center observational study for all patients attending outpatient dermatology clinics at King Abdullah University Hospital (KAUH) in Irbid, Jordan, between January 2022 and March 2024. Approval was obtained through the institutional review board (12/167/2024), which waived the need for consent from patients because the study did not include any therapeutic intervention or procedures. These patients, aged 14 to 36, were exclusively using isotretinoin for severe acne treatment, and we excluded any patient that was using other medications to prevent interference with the menstrual cycle. Patients were selected based on having a regular menstrual cycle, defined as one period every 23–35 days and lasting a week or less, and no personal history of polycystic ovary syndrome (PCOS). Follow-up occurred throughout the treatment period (4.5–8 months) and continued for 2 months post-treatment to monitor any menstrual changes. Comorbidities were not listed due to the age range of our study group. Exclusion criteria included pre-existing menstrual irregularities, pregnancy, use of medications affecting ovarian hormones or causing hirsutism, endocrinopathy, recent isotretinoin treatment, liver dysfunction, contraindications for isotretinoin, unwillingness to use the medication, alcohol consumption, and known comorbidities. In our study, exclusion of polycystic ovary syndrome (PCOS) among participants was rigorously conducted using the Rotterdam Criteria [7], which are internationally recognized for the diagnosis of PCOS. According to these criteria, a diagnosis is confirmed when at least two of the following three conditions are met, after ruling out other causes: (1) oligo and/or anovulation, which is characterized by infrequent or absent menstrual periods, indicating inconsistent ovulation; (2) biochemical and clinical signs of hyperandrogenism, where biochemical indicators include elevated levels of total testosterone (greater than 70 ng/dL), androstenedione (greater than 245 ng/dL), or dehydroepiandrosterone sulfate (greater than 248 mcg/dL), with clinical signs, including severe acne, excessive hair growth in areas where hair is usually minimal or absent, or darkened patches of skin; (3) polycystic ovaries, which are evident from ultrasound findings of 12 or more follicles ranging from 2 to 9 mm in diameter in each ovary or an ovarian volume greater than 10 cubic centimeters. To ensure no participant with PCOS was included, all were screened through detailed medical histories and clinical examinations to check for these criteria. Where PCOS was suspected, further confirmation was sought via biochemical tests and pelvic ultrasound. A comprehensive form was developed to collect data, including medical history, dermatological examination results, and laboratory tests. Variables recorded included age, weight in kilograms, isotretinoin dose in milligrams, treatment duration in months, total accumulative dose, family history, menstrual changes, and onset and normalization of the menstrual irregularities amenorrhea and menorrhagia.

### Statistical Analysis

All data analyses were performed using Jamovi statistical software (version 2.5.4) for Windows. Descriptive measures included means ± standard deviations for continuous data if the normality assumption was not violated according to the Shapiro–Wilk test, and median with interquartile range (IQR) if the assumption was violated. Categorical data were presented by frequencies and percentages (%). Bivariate analyses were conducted comparing patients who experienced irregularities versus those who did not. Continuous data were compared using the Student *t* test in normally distributed variables and the Mann–Whitney *U* test if not normally distributed. Categorical data were compared using the χ^2^ test or the Fisher exact test if 1 cell had an expected count of less than 5. A binary logistic regression analysis was performed to identify the risk factors for having an unfavorable functional outcome in the whole cohort using the enter method. Variables included in the model were chosen based on a separate bivariate analysis, including all variables yielding a *p*-value of <0.1. Nagelkerke *R*^2^ was used as a measure for the goodness of fit. The variables in the model were checked for multicollinearity using the variance inflation factor. Statistical significance was considered at a 2-sided *p*-value of ≤0.05.

## 3. Results

### 3.1. Patient Characteristics

In our study involving 299 individuals undergoing treatment with oral isotretinoin (Table 1), 111 reported alterations in their menstrual cycles, whereas 188 observed no changes. The group experiencing menstrual changes had a median age of 21 years (range 16–34) and a median weight of 58 kg (range 29–87) in contrast to those without changes, whose median age was 22 years (range 14–36) and median weight was 57 kg (range 40–88). Of the 111 reporting changes, 12 (4%) were on a 60 mg dose of isotretinoin, and 99 (33.1%) were on a 40 mg dose; 157 (52.5%) participants did not report any changes. The median treatment duration was 6 months, with a range of 5–8 months for those with menstrual changes and 4.5–7.5 months for those without. The median total cumulative dose was 131 mg/kg (range 120–152) for those experiencing changes versus 133 mg/kg (range 120–150) for those without. Regarding family history of polycystic ovary syndrome (PCOS), 12 (4%) participants with changes had a family history of PCOS and 99 (33.1%) without a family history experienced changes. Conversely, 6 (2%) participants without changes had a family history of PCOS, and 182 (60.9%) without changes had no family history. Ninety-nine (33.1%) reported their menstrual cycle returning to regularity after 2 months, while twelve (4%) said it did not. For those without any menstrual changes, the count was 188 (62.9%). The distribution of menstrual change duration was 7 (2.3%) for one month, 15 (5%) for two months, 53 (17.7%) for three months, 21 (7%) for four months, and 15 (5%) for five months. The types of menstrual changes were primarily amenorrhea (62, 20.7%), oligomenorrhea (46, 15.4%), and menorrhagia (3, 1%).

### 3.2. The Effect of Oral Isotretinoin on the Menstrual Cycle

The median age for those experiencing menstrual alterations was 21 years (range 16–34 years), and for those without changes, it was 22 years (range 14–36 years) (Table 2). The weight median was similar across groups, with a median of 58 kg (range 29–87 kg) for those with menstrual changes and 57 kg (range 40–88 kg) for those without. When comparing isotretinoin dosages, 99 patients (33.1%) on 40 mg and 12 patients (4%) on 60 mg reported menstrual changes, whereas 157 patients (52.5%) on 40 mg and 31 patients (10.4%) on 60 mg did not notice any differences. The median treatment duration was 6 months for both groups, with a slightly wider interquartile range (IQR) for those experiencing changes (5–8 months) compared to those who did not (4.5–7.5 months). A significant finding was the total accumulative dose’s median, which differed between the groups (131 mg/kg for those with changes vs. 133 mg/kg for those without, *p* = 0.008), indicating a possible correlation. Additionally, a family history of polycystic ovary syndrome (PCOS) showed a significant association with menstrual changes (*p* = 0.011). No adverse effects were observed during the study period.

### 3.3. Predictors of Menstrual Irregularities

In the logistic regression analysis (Table 3), the duration of treatment was a significant predictor of menstrual irregularities, with an odds ratio of 5.106 (95% CI: 1.371–19.020, *p* = 0.015), indicating a higher likelihood of menstrual changes with increased treatment duration. The total accumulative dose was also significantly associated with menstrual irregularities (OR = 0.964; 95% CI: 0.939–0.990; *p* = 0.006). A family history of PCOS significantly increased the odds of menstrual irregularities (OR = 3.783; 95% CI: 1.314–10.892; *p* = 0.014). On the other hand, the individual dose of isotretinoin showed a trend towards significance (60 mg vs. 40 mg: OR = 28.803; 95% CI: 0.760–1091.206; *p* = 0.070), suggesting that higher doses may affect menstrual regularity, although this did not reach the conventional threshold for statistical significance. Variables such as age (OR = 0.980; 95% CI: 0.916–1.047; *p* = 0.547) and weight (OR = 0.878; 95% CI: 0.765–1.008; *p* = 0.064) did not demonstrate a significant association with menstrual irregularities in our patient population.

## 4. Discussion

In our study, we aimed to evaluate the effect of oral isotretinoin treatment on the menstrual patterns among female patients in Jordan. We found that patients who had menstrual irregularities were young, with a median age of 21, which is comparable with a previous report in the literature [8]. Approximately 37.1% of our patients with known regular menstrual regular cycles developed irregularities, most commonly amenorrhea (20.7%), after taking oral isotretinoin. These results were consistent with previous reports [9,10]. The mechanism by which oral isotretinoin can lead to menstrual cycle irregularities is unknown and poorly understood. Recent evidence has concluded that isotretinoin interacts with several biological and hormonal pathways [9,11,12]. Interestingly, several lines of evidence suggested that isotretinoin is associated with psychiatric side effects through its influence on retinoid signaling pathways, which are involved in regulating gene expression via the retinoic acid receptors [13]. This shifts to the current theory that women who suffer from severe acne are more likely to experience stress and menstrual cycle irregularities [14,15]. From this relationship, we believe that stress is a key regulator in irregularities accompanying isotretinoin therapy. A study performed by Molla et al. [15] found a significant relationship between anxiety and depression scores in patients with acne. Our study did not find any significant differences in age, weight, dose of the medication, and duration of the treatment between the study groups. Conversely, the total accumulative dose of isotretinoin and a family history of PCOS were significantly correlated with menstrual irregularities. These findings suggest that the individual characteristics and treatment specifics of each patient play a crucial role in the side effects experienced during isotretinoin therapy. The significant connection between total accumulative dose and menstrual changes shows that the effect of isotretinoin on menstrual regularity is a dose dependent. This stands with the hypothesis that more menstrual cycle changes with higher accumulative doses may be due to isotretinoin’s effect on ovarian function and alterations in hormone levels [10,16,17]. In our logistic regression analysis, we sought to identify significant predictors of menstrual irregularities among female patients undergoing oral isotretinoin treatment. The analysis revealed that the duration of isotretinoin treatment and the total accumulative dose were significant predictors of menstrual changes. Specifically, patients with longer treatment durations were more likely to experience menstrual irregularities, suggesting a possible cumulative effect of the drug over time on menstrual health. Moreover, the total accumulative dose of isotretinoin presented a significant association with menstrual irregularities. This finding supports a dose-dependent relationship and raises concerns about the implications of higher cumulative dosages on menstrual cycle regularity. Interestingly, a family history of polycystic ovary syndrome (PCOS) emerged as a significant factor, indicating that patients with such a history were more likely to report menstrual irregularities while on isotretinoin therapy. This could suggest a genetic predisposition that may interact with the drug’s effects on menstrual cycles. While not reaching conventional levels of statistical significance, the odds ratio for a higher isotretinoin dose (60 mg vs. 40 mg) indicated a considerable increase in the likelihood of menstrual irregularities. This may suggest that higher doses of isotretinoin may have a disproportionate impact on menstrual cycle regularity, although this needs to be interpreted with caution due to the wide confidence interval, which includes unity. On the contrary, age and weight did not show a statistically significant impact in our model. These findings indicate that while these factors might influence individual susceptibility, they do not universally predict menstrual irregularities across the population studied. The absence of statistical significance in age and weight as predictors could reflect a complex interplay between hormonal regulation and individual variability. These factors, although not significant in isolation, should not be disregarded as they may still play a contributory role in the broader context of isotretinoin’s effects on reproductive health.

To our knowledge this is the first study in Jordan that studied menstrual cycle irregularities in patients with oral isotretinoin treatment for acne. Our strength lies in the study population being from a tertiary hospital in Jordan. This allowed us to include patients from different geographical regions in Jordan, thereby insuring the generalizability of our results. However, our study results should be interpreted with caution in the context of several limitations. The absence of a control group limited our ability to directly assess whether the menstrual cycle changes are directly attributed to isotretinoin or to other physiological changes. Additionally, psychological factors such as depression and anxiety are well-known causes of menstrual cycle irregularities, and they were not investigated in this study. This omission represents a significant limitation as these factors could confound the relationship between isotretinoin and menstrual changes. Furthermore, the exclusion of patients with any prior menstrual irregularities or other significant health issues may further limit the applicability of the findings to the general population of isotretinoin users. Finally, we did not evaluate changes in patient weight or investigate other potential confounders such as stress or external health conditions like COVID-19, which might influence menstrual regularity independent of treatment. Due to these factors, future research should focus on several key areas. Conducting longitudinal studies with control groups could isolate the drug’s specific effects, while incorporating psychological assessments would help differentiate the psychological impacts from the physiological effects of isotretinoin. Exploring the hormonal mechanisms through hormonal profiling and investigating the dose-response relationship would provide deeper insights into how different dosages affect menstrual regularity. Additionally, studies across multiple centers with diverse populations would enhance the generalizability of the findings. Research into the genetic predispositions that may influence susceptibility to side effects could facilitate personalized treatment plans. Finally, extended post-treatment follow-up would be crucial for understanding the long-term effects of isotretinoin on menstrual health and determining the reversibility of any changes. This study highlights important clinical implications regarding the administration of oral isotretinoin and its effects on menstrual irregularities in patients with acne, which could significantly enhance current dermatological practices and patient management strategies. Significant predictors such as treatment duration, total accumulative dose, and a family history of PCOS not only underscore the necessity for clinicians to adopt a more personalized approach to prescribing isotretinoin but also prompt a reevaluation of patient monitoring protocols. The finding that longer treatment durations and higher cumulative doses are associated with increased risks of menstrual irregularities suggests that clinicians should consider these factors when determining the regimen for their patients. This might involve initiating treatment at lower doses and increasing the dose gradually or possibly integrating more frequent menstrual health assessments into the treatment protocol, especially for patients with a known family history of PCOS. Furthermore, given that most affected patients experienced a return to normal menstrual cycles post-treatment, clinicians should reassure patients about the potential reversibility of these side effects, which could play a crucial role in patient compliance and satisfaction. However, the significant impact of these factors also highlights the urgent need for tailored patient education and counseling about the possible side effects of isotretinoin on menstrual health, empowering patients to report changes promptly.

Considering these results, healthcare providers should consider these risk factors when prescribing isotretinoin. Patients with a long duration of treatment, higher cumulative doses, or a family history of PCOS should be monitored closely for menstrual irregularities [5].

## 5. Conclusions

Our investigation into the impact of oral isotretinoin on menstrual patterns in female patients in Jordan has yielded significant insights into the relationship between isotretinoin therapy and menstrual irregularities. We discovered that approximately 37.1% of patients with previously regular menstrual cycles experienced changes, notably amenorrhea, during treatment. The study identified several critical predictors of menstrual irregularities, including the duration of treatment and the total accumulative dose of isotretinoin, as well as a family history of polycystic ovary syndrome (PCOS). These factors suggest a dose-dependent effect of isotretinoin on menstrual regularity, emphasizing the importance of monitoring and potentially adjusting treatment protocols for patients at higher risk of experiencing these side effects.

## Figures and Tables

**Table 1:** Baseline characteristics and menstrual changes in female patients undergoing oral isotretinoin therapy.

Variable, *n* (%)		Changes in Menses	
	All Patients (299)	Yes (111)	No (188)
Age (years, median (IQR))	22 (5)	21 (6)	22 (4.25)
Weight (kg, median (IQR))	58 (13)	58 (29–87)	57 (40–88)
Dose (mg), *n* (%)			
40	256 (85.6%)	99 (33.1)	157 (52.5)
60	43 (14.4%)	12 (4)	31 (10.4)
Duration of treatment (months, median (IQR))	6 (0.5)	6 (5–8)	6 (4.5–7.5)
Total accumulative dose (mg/kg, median (IQR))	131 (18.2)	131 (120–152)	133 (120–150)
Family history of PCOS, *n* (%)			
No	282 (94)	99 (33.1)	182 (60.9)
Yes	18 (6)	12 (4)	6 (2)
Menses back to regular after 2 months, *n* (%)			
No change	188 (62.9)	0	188 (62.9)
No	12 (4)	12 (4)	0
Yes	99 (33.1)	99 (33.1)	0
Months of change, *n* (%)			
0	188 (62.9)	0	188
1	7 (2.3)	7 (2.3)	0
2	15 (5)	15 (5)	0
3	53 (17.7)	53 (17.7)	0
4	21 (7)	21 (7)	0
5	15 (5)	15 (5)	0
Type of change, *n* (%)			
Amenorrhea	62 (20.7)	62 (20.7)	0
Oligomenorrhea	46 (15.4)	46 (15.4)	0
Menorrhagia	3 (1)	3 (1)	0

Abbreviations: PCOS, polycystic ovary syndrome.

**Table 2:** Comparative analysis of menstrual changes in patients on oral isotretinoin treatment.

Variable, *n* (%)	Changes in Menses		
	Yes (111)	No (188)	*p*-Value
Age (years, median (IQR))	21 (16–34)	22 (14–36)	0.314
Weight (kg, median (IQR))	58 (29–87)	57 (40–88)	0.175
Dose (mg)			0.232
40	99 (33.1)	157 (52.5)	
60	12 (4)	31 (10.4)	
Duration of treatment (months, median (IQR))	6 (5–8)	6 (4.5–7.5)	0.198
Total accumulative dose (mg/kg, median (IQR))	131 (120–152)	133 (120–150)	0.008
Family history of PCOS			0.011
No	99 (33.1)	182 (60.9)	
Yes	12 (4)	6 (2)	

Abbreviations: PCOS: polycystic ovary syndrome, IQR: interquartile range.

**Table 3:** Binary logistic regression identifying predictors of menstrual irregularities after oral isotretinoin treatment.

Predictors		95% Confidence Interval		*p* Value
	Odds Ratio	Lower	Upper	
Age	0.980	0.916	1.047	0.547
Weight (kg)	0.878	0.765	1.008	0.064
Duration of treatment (months)	5.106	1.371	19.020	0.015
Total accumulative dose (mg/kg)	0.964	0.939	0.990	0.006
Family history of PCOS:				
No	1 (Reference)	1 (Reference)	1 (Reference)	1 (Reference)
Yes	3.783	1.314	10.892	0.014
Dose (mg)				
40	1 (Reference)	1 (Reference)	1 (Reference)	1 (Reference)
60	28.803	0.760	1091.206	0.070

*p*-value less than 0.05 indicates statistical significance.

## Data Availability

Data used in the article are available on request from the corresponding author.
